# Laser Vaporization of Intracoronary Thrombus and Identifying Plaque Morphology in ST-Segment Elevation Myocardial Infarction as Assessed by Optical Coherence Tomography

**DOI:** 10.1155/2021/5590109

**Published:** 2021-07-28

**Authors:** Yuki Yamanaka, Yoshihisa Shimada, Daisuke Tonomura, Kazunori Terashita, Tatsuya Suzuki, Kentaro Yano, Satoshi Nishiura, Masataka Yoshida, Takao Tsuchida, Hitoshi Fukumoto

**Affiliations:** Cardiovascular Center, Shiroyama Hospital, 2-8-1 Habikino, Habikino 583-0872, Japan

## Abstract

**Objectives:**

We evaluated the thrombus-vaporizing effect of excimer laser coronary angioplasty (ELCA) in patients with ST-segment elevation myocardial infarction (STEMI) by optical coherence tomography (OCT).

**Background:**

Larger intracoronary thrombus elevates the risk of interventional treatment and mortality in patients with STEMI.

**Methods:**

A total of 92 patients with STEMI who presented within 24 hours from the onset and underwent ELCA following manual aspiration thrombectomy (MT) were analyzed.

**Results:**

The mean baseline thrombolysis in myocardial infarction flow grade was 0.4 ± 0.6, which subsequently improved to 2.3 ± 0.7 after MT (*p* < 0.0001) and 2.7 ± 0.5 after ELCA (*p*=0.0001). The median residual thrombus volume after MT was 65.7 mm^3^, which significantly reduced to 47.5 mm^3^ after ELCA (*p* < 0.0001). Plaque rupture was identified by OCT in only 22 cases (23.9%) after MT, but was distinguishable in 36 additional cases after ELCA (total: 58 cases; 63.0%). Ruptured lesions contained a higher proportion of red thrombus than nonruptured lesions (75.9% vs. 43.3%, *p*=0.001). Significantly larger thrombus burden after MT (69.6 mm^3^ vs. 56.3 mm^3^, *p* < 0.05) and greater thrombus reduction by ELCA (21.2 mm^3^ vs. 11.8 mm^3^, *p* < 0.01) were observed in ruptured lesions than nonruptured lesions.

**Conclusions:**

ELCA effectively vaporized intracoronary thrombus in patients with STEMI even after MT. Lesions with plaque rupture contained larger thrombus burden that was frequently characterized by red thrombus and more effectively reduced by ELCA.

## 1. Introduction

Intracoronary thrombus burden is a major determinant of adverse clinical outcome in patients with ST-segment elevation myocardial infarction (STEMI). Larger thrombus burden limits the success of percutaneous coronary intervention (PCI) for STEMI, as it increases the rate of procedural complications, such as distal embolization and no reflow phenomenon. It is also associated with worse microvascular dysfunction and greater myocardial damage, thereby significantly affecting mortality [1, 2]. Therefore, theoretically, reduction of intracoronary thrombus at the culprit lesion improves the outcome of primary PCI and reduces the mortality rate in patients with STEMI.

However, routine thrombus aspiration remains of uncertain value in primary PCI for STEMI. Several randomized studies have demonstrated that manual aspiration thrombectomy (MT) for STEMI prevents the occurrence of the no reflow phenomenon and distal embolization, resulting in better myocardial reperfusion and reduced myocardial infarct size [3–5]. In contrast, larger randomized clinical trials have not shown the clinical benefit of the routine aspiration strategy compared with standard PCI [2, 6]. These contradictory findings may be attributed to insufficient removal of thrombus using current aspiration thrombectomy devices. Several optical coherence tomography (OCT) studies demonstrated that MT had no impact on the reduction of thrombus burden in STEMI, and substantial residual thrombus was observed even after MT at the culprit segment [7, 8].

Excimer laser is a unique revascularization device based on the effect of a pulsed ultraviolet (xenon-chloride) laser that directly targets and vaporizes the thrombus. Excimer laser coronary angioplasty (ELCA) has been shown to be safe and effective in acute ischemic-thrombotic coronary syndromes by successful vaporization of intracoronary thrombus at the target lesion [9–12]. Therefore, ELCA may be a useful adjunctive strategy with the potential to further reduce the thrombotic burden which poorly responds to MT. The purpose of this study was to evaluate the efficacy of ELCA following MT in patients with STEMI, by measuring the thrombus burden before and after ELCA using OCT.

## 2. Methods

ELCA for thrombotic coronary lesions has been utilized in our hospital since November 2015. This was a retrospective study of consecutive patients who presented with STEMI within 24 hours after the onset and underwent ELCA under OCT guidance at our hospital. STEMI was defined as continuous chest pain that lasted >20 min, electrocardiogram showing new ST-segment elevation ≥0.2 mV in at least two contiguous precordial leads or ≥0.1 mV in at least two contiguous limb leads, and angiographic identification of a coronary thrombotic stenosis or occlusion. This study was approved by the ethics committee of our hospital and conducted in accordance with the principles of the Declaration of Helsinki. Written informed consent was provided by all patients.

Heparin was administered intravenously with an initial bolus of 5,000 U, followed by periodic shots to maintain an activated clotting time between 200 and 300 s during the procedure. Dextran of mean molecular weight 40 kDa (50–100 mL of 10% dextran-40 per hour) was also used during the procedure to prevent thromboembolic complications. After initial coronary angiography, MT was first performed using a manual thrombectomy device (Thrombuster III GR; Kaneka Corp., Osaka, Japan or Export Advance; Medtronic, Dublin, Ireland) and repeated at least thrice until there was no visible thrombotic material in the aspirate. ELCA was subsequently performed using a pulsed xenon-chloride excimer laser system with a wavelength of 308 nm; pulse duration of 135 ns; and output of 175 mJ/pulse (CVX-300P; Royal Philips, Amsterdam, Netherlands). The laser atherectomy catheters (ELCA™ Coronary Laser Atherectomy Catheter; Royal Philips) were available in sizes of 0.9, 1.4, 1.7, and 2.0 mm, and the default energy parameters were set at a fluence of 45 mJ/mm^2^ and a repetition rate of 25 Hz. The size of the catheter, maximum fluency, and repetition rate (up to 60 mJ/mm^2^ and 40 Hz, respectively) and the number of passes were at the operator's discretion. The laser vaporizing manner employed in this study was previously described [9]. The procedure was followed by balloon dilatation and finalized with drug-eluting stent implantation or drug-coated balloon utilization. All patients received loading doses of oral antiplatelet agents immediately after the procedure, consisting of aspirin (200 mg) and P2Y12 inhibitor (clopidogrel 300 mg or prasugrel 20 mg) if they were not pretreated with a dual antiplatelet therapy.

A frequency-domain OCT imaging catheter system (Dragonfly OPTIS/ILUMIEN OPTIS OCT Imaging System; Abbott Vascular Inc., Santa Clara, CA, USA) was used in this study. The OCT imaging acquisition was performed after MT, ELCA, and final ballooning, with a pullback speed of 18 mm/s and acquisition of 180 frames/s. Intracoronary administration of isosorbide dinitrate (200–500 mg) was performed prior to each pullback. The acquired OCT raw data were stored and exported in digital format for offline analyses using a dedicated offline review system with a semiautomated contour-detection software (Abbott Vascular Inc). All OCT images were analyzed by two experienced investigators (Y.Y. and D.T.). In case of any discordance between the observers, a consensus reading was obtained from a third investigator (Y.S.).

The entire length of the atherothrombotic lesion plus 5 mm proximal and distal reference segments were included in the analysis. Intracoronary thrombus was defined as an irregular mass attached to the luminal surface or floated from the vessel wall and characterized according to the signal characteristics [1, 13]. The predominant type of thrombus was categorized according to the signal characteristics: highly backscattering with high attenuation (red thrombus), less backscattering and homogeneous with low attenuation (white thrombus), or a mixture of both (mixed thrombus) [13, 14]. Thrombus and lumen area were measured by planimetry at 1 mm intervals with the automatic lumen contour and supplementary manual correction, and the thrombus and lumen volume were calculated by multiplying the areas in each frame by the number of frames [1, 13, 15]. The distance between the most distal and proximal frames that showed intraluminal material suggestive of thrombus defined the thrombus length [13].

The culprit lesion was categorized into four groups: plaque rupture, plaque erosion, calcified nodule, and unidentified [1, 14]. Plaque rupture was defined by the presence of fibrous-cap discontinuity with a cavity in the plaque. Plaque erosion was defined by the presence of an attached thrombus overlying an intact and visualized plaque or irregular luminal surface with no evidence of fibrous-cap disruption. Calcified nodule was defined by the presence of nodular protruding calcium or luminal surface disruption over a calcified plaque associated with the thrombus. The culprit lesions, unable to be recognized based on the aforementioned criteria by the three observers, were classified as unidentified.

Normally distributed continuous variables are expressed as mean ± standard deviation, while nonnormally distributed continuous variables are presented as median and interquartile range. Categorical variables are expressed as counts and percentages. Repeated-measures analysis of variance with post hoc Tukey's honest significant difference test was applied to compare serial parameters after each procedure. The Mann–Whitney *U* test or Pearson's chi-squared test was applied to examine differences between the lesions with ruptured plaque and those with nonruptured plaque. A *p* value <0.05 was considered statistically significant. All statistical analyses were performed using the JMP statistical software (version 13.2; SAS Institute, Cary, NC, USA).

## 3. Results

**Figure 1:**
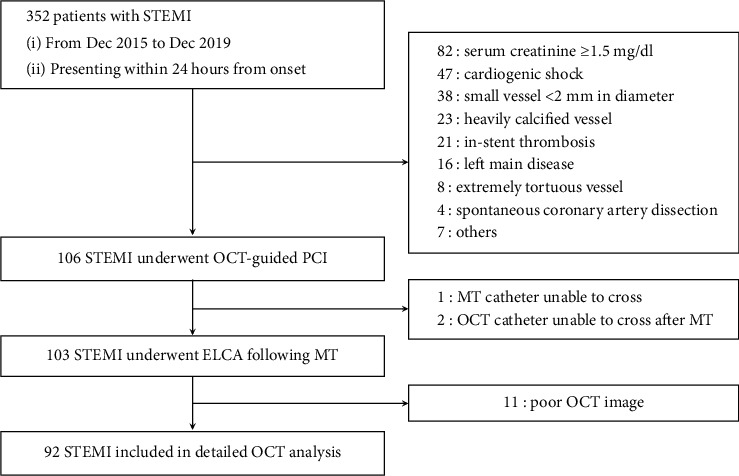
Study flow chart. ELCA, excimer laser coronary angioplasty; MT, manual aspiration thrombectomy; OCT, optical coherence tomography; and STEMI, ST-segment elevation myocardial infarction.

From December 2015 to December 2019, 352 consecutive patients with STEMI who presented within 24 h after the onset of symptoms underwent emergent PCI at our hospital. OCT was not attempted under the following conditions: patients with cardiogenic shock (*n* = 47); STEMI caused by in-stent thrombosis (*n* = 21) or spontaneous coronary artery dissection (*n* = 4); impaired kidney function (serum creatinine ≥1.5 mg/dl; *n* = 82); left main disease (*n* = 16); small-vessel disease (<2 mm in diameter; *n* = 38); extremely tortuous vessels (*n* = 8); heavily calcified vessels (*n* = 23); or other conditions the physicians considered inappropriate (*n* = 7). Therefore, 106 patients underwent OCT-guided PCI with the intention to perform MT followed by ELCA (Figure 1). Patients in whom it was not possible to advance the manual aspiration catheter (*n* = 1) or OCT catheter (*n* = 2) distal to the culprit lesion before ELCA or those with poor OCT image quality (*n* = 11) were excluded from this study. Finally, a total of 92 lesions in 92 patients with STEMI who underwent aspiration thrombectomy followed by ELCA of the infarct-related artery were analyzed in this study.

**Figure 2:**
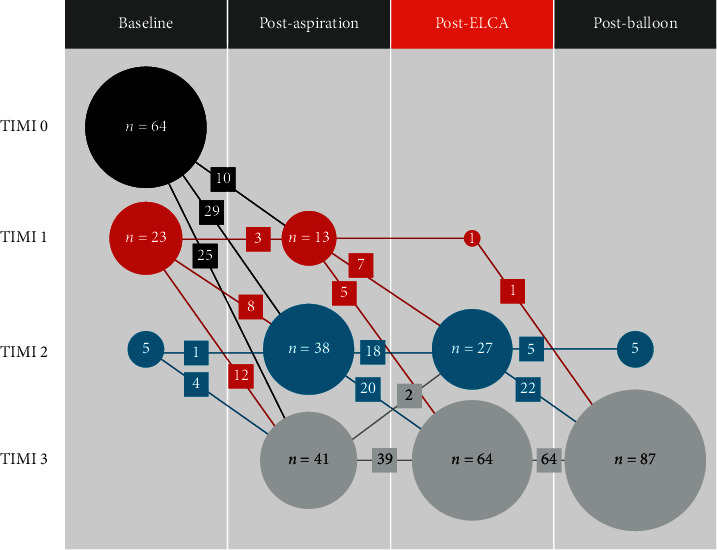
TIMI flow grade and the number of the patients after each of the procedures. TIMI, thrombolysis in myocardial infarction.

Baseline patient, lesion, and procedural characteristics are shown in Tables 1 and 2. A laser size of 1.7 mm was used in >80% of cases, mainly because it is the maximum laser size compatible with a 6Fr guide catheter. The excimer laser catheter was successfully crossed distal to the culprit lesion in all cases without any balloon predilatation. The mean baseline thrombolysis in myocardial infarction (TIMI) flow grade on initial angiography was 0.4 ± 0.6. The TIMI flow grade was significantly improved by MT to 2.3 ± 0.7 (*p* < 0.0001) compared to that at baseline and subsequently improved by ELCA to 2.7 ± 0.5 (*p*=0.0001) compared to that at post MT (Figure 2). None of the cases developed ELCA-induced perforation, angiographical distal embolization, or flow-limiting dissection. ELCA-induced TIMI flow deterioration was observed in two cases (2.2%); however, no visible distal embolization was detected. TIMI 3 flow was achieved in 87 of 92 patients (94.6%) after adjunctive balloon angioplasty and 90 of 92 patients (97.8%) after final devices (drug-eluting stent or drug-coated balloon).

The results of the OCT analysis are shown in Table 3. Compared with those after MT, ELCA significantly reduced thrombus volume (from 62.8 mm^3^ to 46.2 mm^3^, *p* < 0.0001), resulting in larger lumen volume (from 70.0 mm^3^ to 87.7 mm^3^, *p* < 0.0001) and minimum lumen area (from 1.4 ± 0.7 mm^2^ to 2.4 ± 0.9 mm^2^, *p* < 0.0001) (after MT and after ELCA, respectively).

**Figure 3:**
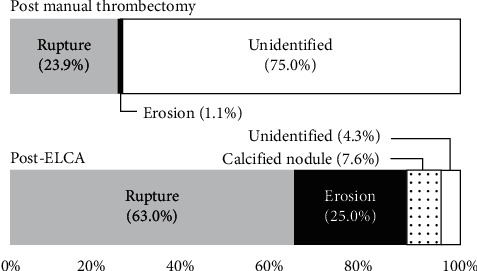
OCT identification of lesion morphology after MT and ELCA. Lesion morphology was not identifiable in the majority of cases even after MT because of the residual thrombus occupying the lumen. Vaporization of the thrombus by ELCA facilitated the identification of plaque morphology using OCT. OCT, optical coherence tomography; MT, manual aspiration thrombectomy; and ELCA, excimer laser coronary angioplasty.

**Figure 4:**
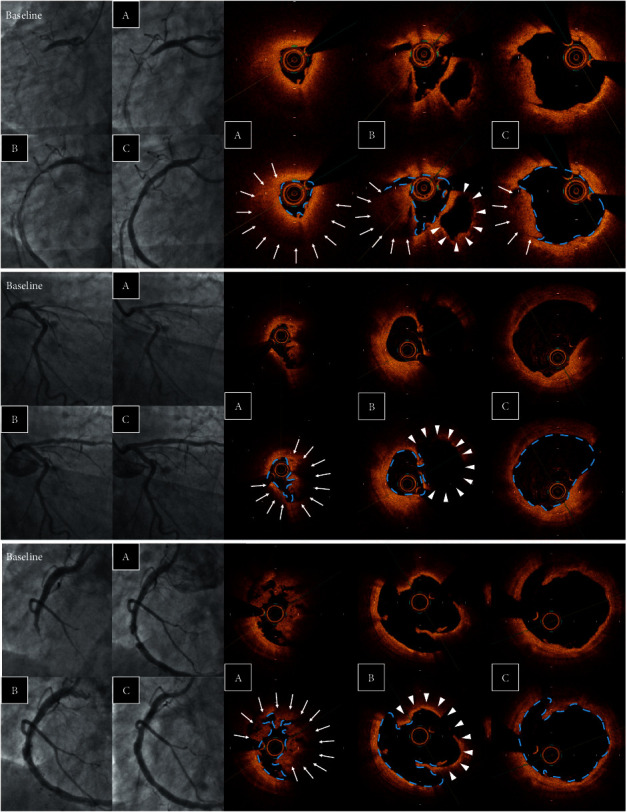
Examples of plaque rupture. Angiographic and OCT images after (A) MT, (B) ELCA, and (C) ballooning. Laser vaporization of the thrombus (arrow) enabled the identification of the ruptured cavity (arrow head). ELCA, excimer laser coronary angioplasty; MT, manual aspiration thrombectomy; and OCT, optical coherence tomography.

**Figure 5:**
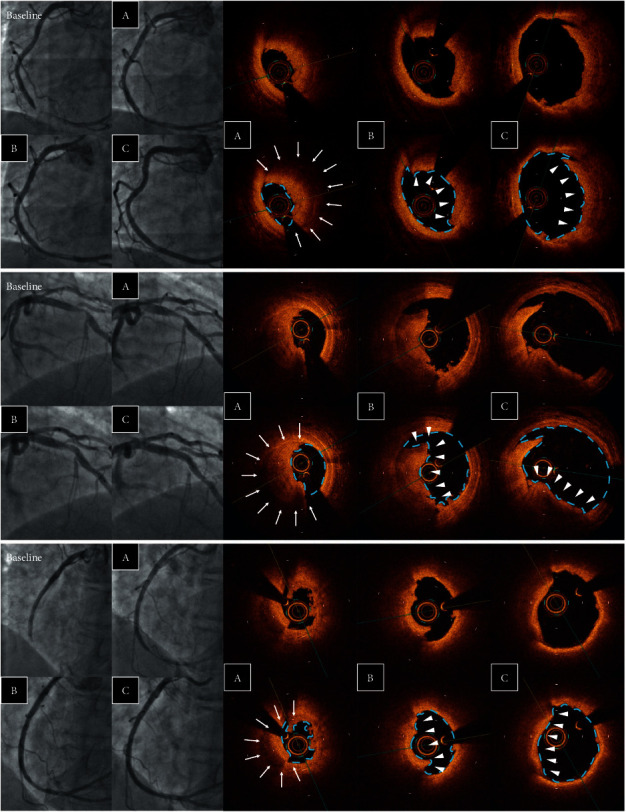
Examples of plaque erosion. Angiographic and OCT images after (A) MT, (B) ELCA, and (C) ballooning. Laser vaporization of the thrombus (arrow) clarified the identification of the attached thrombus overlying the eroded plaque (arrow head). ELCA, excimer laser coronary angioplasty; MT, manual aspiration thrombectomy; and OCT, optical coherence tomography.

**Figure 6:**
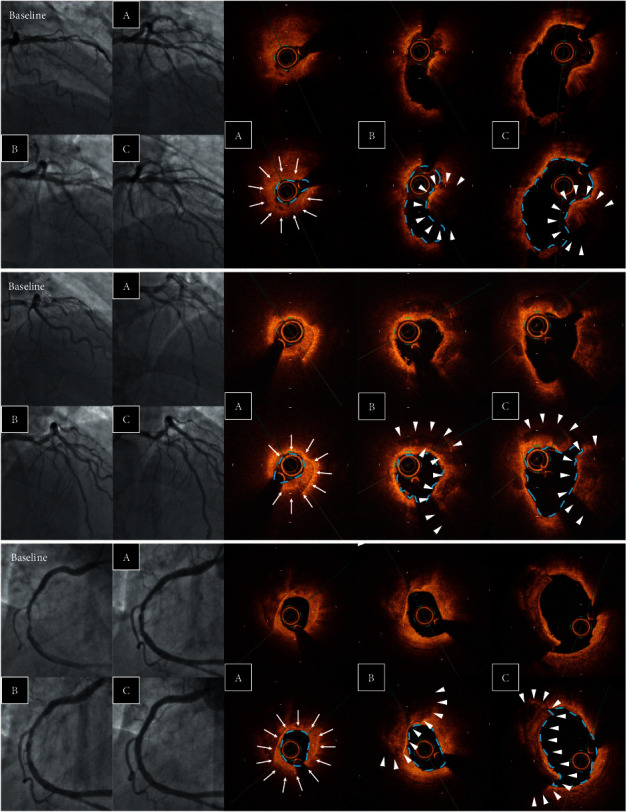
Examples of calcium nodules. Angiographic and OCT images after (A) MT, (B) ELCA, and (C) ballooning. Laser vaporization of the thrombus (arrow) clarified the identification of nodular protruding calcium (arrow head). ELCA, excimer laser coronary angioplasty; MT, manual aspiration thrombectomy; and OCT, optical coherence tomography.

**Figure 7:**
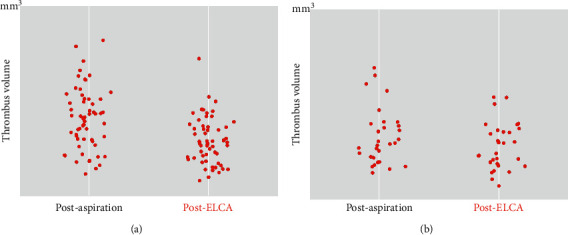
Scatter plots of thrombus volume after manual aspiration thrombectomy and laser vaporization. ELCA, excimer laser coronary angioplasty. (a) Ruptured plaque. (b) Nonruptured plaque.

Using the OCT images after MT, plaque rupture at the culprit lesion was identified in only 22 of 92 cases (23.9%), but was successfully distinguished in another 36 cases (total 58 cases: 63.0%) after ELCA (Figures 3 and 4). Among the other cases, OCT after ELCA demonstrated the morphology of plaque erosion in 23 cases (25.0%) (Figure 5) and calcified nodule in seven cases (7.6%) (Figure 6), whereas the remaining four cases (4.3%) were unidentifiable. Red thrombus was observed more frequently in lesions with ruptured plaque than those with nonruptured plaque (75.9% vs. 43.3%, respectively, *p*=0.001) (Table 4). Although the residual thrombus burden after MT was larger in lesions with ruptured plaque (69.6 mm^3^ vs. 56.3 mm^3^, respectively, *p* < 0.05), the vaporizing effect by ELCA on reducing the thrombus burden was more pronounced in lesions with ruptured plaque than those with nonruptured plaque (21.2 mm^3^ vs. 11.8 mm^3^, respectively, *p* < 0.01) (Figure 7).

## 4. Discussion

Thrombus burden at the culprit lesion remains an important risk factor for cardiac damage and mortality following primary PCI in patients with STEMI [1, 2]. Mechanical removal of the occlusive thrombus appears to be a logical option for the treatment of these highly thrombotic lesions, and several clinical trials demonstrated that MT during primary PCI resulted in a lower risk of distal embolization and no reflow phenomenon, better ST-segment resolution, preserved microvascular integrity, lower myocardial damage, and reduced mortality rate [3–5]. However, recent large randomized trials and a historical cohort study failed to show an additional benefit of routine thrombus aspiration in patients with STEMI [2, 6]. This may be attributed to insufficient removal of the thrombus at the culprit lesion by the currently available MT devices. Previous OCT studies demonstrated that MT did not significantly reduce the thrombus burden at the culprit lesions versus lesions without MT [7, 8]. In our study, we also observed a substantial amount of OCT-identified residual thrombus even after repeated MT.

Our study demonstrated that ELCA additionally reduced thrombus burden at the culprit lesion and restored luminal flow space, as measured using OCT even after MT. In addition, ELCA significantly enhanced the restoration of anterograde TIMI flow of the infarct-related artery without distal embolism. Previous reports have shown ELCA to be feasible and efficient in primary PCI for STEMI, demonstrating improvement in TIMI flow, TIMI frame count, and TIMI myocardial blush grade [9–12]. The ELCA effect is based on three unique mechanisms of a pulsed xenon-chloride laser (photochemical, photothermal, and photomechanical), and its thrombus-vaporizing effect may exceed the simple mechanical effect of a manual aspiration catheter on reducing the thrombus burden [16].

The products of in vitro excimer laser thrombolysis are reported to be <10 micron in size [17], and previous clinical studies reported that ELCA achieved a higher rate of tissue-level reperfusion (myocardial blush grade, ST-segment resolution) than MT and balloon angioplasty in patients with STEMI [18, 19]. However, even invisible microdebris may cause microcirculatory impairment during ELCA, such as during rotational coronary atherectomy, resulting in slow or no reflow phenomenon. In addition, microcirculatory impairment can occur more frequently in the setting of acute myocardial infarction because of reperfusion injury, platelet aggregation, vasospasm, or tissue edema. In this study, ELCA-induced TIMI flow deterioration was observed in two cases (2.2%). We assume that this might be attributable to the microcirculatory disorder, albeit no visible distal embolization was detected.

More thorough removal of thrombus by ELCA may be beneficial, particularly to patients with large thrombus burden. In our study, the effect of ELCA on thrombus dissolution was more pronounced in lesions with extensive thrombus. This finding is in line with previous studies which showed significantly higher acute laser gain in lesions with larger thrombus burden [9, 12]. However, estimating thrombus burden by angiography alone is often difficult, and a previous study reported that the angiographic thrombus grade does not correlate with intracoronary thrombus volume as measured by OCT [20]. OCT is currently the most reliable method of thrombus quantification *in vivo* [13, 15]. Therefore, it may potentially stratify patients with high thrombus burden who further benefit from thrombus dissolution therapy, such as ELCA.

Vaporizing thrombus by ELCA may assist in understanding the pathophysiology of the culprit lesion in STEMI. In this study, a significant amount of residual thrombus hampered the assessment of the underlying plaque morphology in most cases even after MT. This was mainly because of the fundamental limitation of the OCT signal being unable to penetrate red cell-rich thrombus. Even with its high resolution of OCT imaging, the majority of plaque ruptures were identifiable after thrombus vaporization by ELCA. Previous studies have reported that the exposed necrotic core of ruptured plaque is highly thrombogenic and contains larger amounts of red thrombus compared with other plaque phenotypes [21, 22]. Our study demonstrated that adequate removal of sizable red thrombus, which causes rapid attenuation of the OCT signal, may be required for more precise detection of ruptured plaque in the culprit lesion in STEMI.

Aggressive thrombus removal and understanding the morphology of the culprit lesion may potentially lead to better management of the therapeutic strategies. In STEMI, the pathological culprit lesion is often located proximal or distal to the angiographical occlusion site [23]; therefore, incomplete stent coverage of the ruptured site is relatively common [24]. This insufficient lesion coverage appears to have a substantial clinical impact because the stent-uncovered ruptured plaque often exhibits chronic lumen reduction during the healing process [25]. In addition, although the rupture site appears to be adequately covered by the stent, chronic thrombus dissolution over time may result in late stent malapposition, which is significantly more frequent in ruptured plaque [26]. On the other hand, nonruptured plaque, such as lesions with plaque erosion, usually contains less thrombus burden and can be safely managed by oral dual antiplatelet therapy alone without intracoronary stenting [27]. Therefore, *in vivo* identification of culprit plaque morphology by reducing the intracoronary thrombus burden with ELCA may assist in developing an interventional approach for patients with STEMI.

### 4.1. Limitations

This was a single-center and nonrandomized study wherein ELCA was employed in all lesions. A further randomized study comparing PCI for STEMI with and without ELCA is required to identify the detailed benefits of ELCA. None of the patients were pretreated with a glycoprotein IIb/IIIa inhibitor, as this agent is not available in Japan. Potent antiplatelet or antithrombotic therapies may have influenced the efficacy of ELCA in vaporizing the intracoronary thrombus. The thrombus clot at the site of culprit lesion may, to some extent, embolize distally during insertion of the OCT or the laser catheter. Although the OCT catheter is smaller (3.0 Fr at the distal shaft) and the laser catheter is similar (5.3 Fr; 1.7c) in size to the manual aspiration catheter (5.1 Fr), the possibility that the reduced thrombus volume was simply because of insertion of any catheter into the coronary artery cannot be ruled out; this may have potentially altered the quantitative analysis of the thrombus volume. The number of patients included in the present study was relatively small, especially for assessing the distribution of culprit lesion morphology in STEMI. However, the sample size of 92 patients treated with ELCA and assessed by OCT to evaluate its efficacy is the largest population analyzed with this technique thus far. Finally, the finding of more plaque rupture after ELCA may not only reflect greater clearance of thrombus but also be related to laser-induced intimal injury. Although previous studies demonstrated that atheroma mass is minimally ablated by ELCA and that ELCA-induced channels are relatively smooth without relevant histologic damage [28, 29], ELCA may have caused vascular injury and affected the identification of the lesion morphology.

## 5. Conclusions

In patients with STEMI, ELCA was feasible and effective in additionally reducing the intracoronary thrombus burden and creating a larger blood flow area even after MT. Moreover, the vaporization of the thrombus by ELCA also facilitated the identification of plaque morphology using OCT. Lesions with plaque rupture contained a larger thrombus burden that was frequently characterized by red thrombus and was more effectively reduced by ELCA compared to those with nonruptured plaque.

## Figures and Tables

**Table 1:** Patient characteristics.

Variable	Value
Number of patients	92
Age (years)	63.5 ± 12.6
Male sex	77 (83.7%)
Hypertension	59 (64.1%)
Diabetes mellitus	38 (41.3%)
Hemoglobin A1c (%)	6.5 ± 1.4
Dyslipidemia	54 (58.7%)
LDL-cholesterol (mg/dL)	126.4 ± 38.8
Current smoking	45 (48.9%)
Serum creatinine (mg/dL)	0.85 ± 0.78
Prior MI	1 (1.1%)
Prior PCI	1 (1.1%)
Symptom onset to hospital arrival (h)	5.5 ± 5.5
Baseline CPK (IU/L)	460 ± 762
Baseline CPK-MB (IU/L)	43 ± 65
Peak CPK (IU/L)	2,868 ± 2,391
Peak CPK-MB (IU/L)	272 ± 239

Data are presented as *n* (%) or mean ± standard deviation. CPK = creatine phosphokinase; CPK-MB = creatine phosphokinase myocardial band; LDL = low-density lipoprotein; MI = myocardial infarction; PCI = percutaneous coronary intervention.

**Table 2:** Lesion and procedural characteristics.

Variable	Value
Culprit lesion location	
LAD	41 (44.6%)
LCx	11 (12.0%)
RCA	40 (43.5%)

Angiographic parameters	
Reference diameter (mm)	2.98 ± 0.51
Lesion length (mm)	19.0 ± 4.0
Minimal lumen diameter (mm)	0.15 ± 0.26
Diameter stenosis (%)	94.6 ± 9.1

Initial TIMI flow grade	
TIMI 0	64 (69.6%)
TIMI 1	23 (25.0%)
TIMI 2	5 (5.4%)

Access site and the size of guide catheter	
Transradial (6 Fr)	84 (91.3%)
Transfemoral (6 Fr/8 Fr)	7/1 (7.6%/1.1%)

Laser catheter used	
1.4 c	15 (16.3%)
1.7 c	74 (80.4%)
1.7 e	2 (2.2%)
2.0 c	1 (1.1%)
Maximum energy fluency (J)	56.9 ± 6.2
Maximum repetition rate (Hz)	27.7 ± 5.7
Laser pulses delivered	2,338 ± 1,380

Final device	
DCB	87 (94.6%)
Size of DCB (mm)	3.28 ± 0.46
Total length of DCB (mm)	28.8 ± 12.0
DES	5 (5.4%)
Size of DES (mm)	3.35 ± 0.22
Total length of DES (mm)	34.8 ± 12.1

DCB = drug-coated balloon; DES = drug-eluting stent; LAD = left anterior descending artery; LCx = left circumferential artery; RCA = right coronary artery; TIMI = thrombolysis in myocardial infarction.

**Table 3:** Results of optical coherence tomography results.

Variable	Value
Thrombus volume (mm^3^)	
After manual thrombectomy	62.8 (41.4–85.4)
After ELCA	46.2 (28.3–62.3)
After ballooning	13.3 (4.0–27.7)

Luminal volume (mm^3^)	
After manual thrombectomy	70.0 (51.8–86.7)
After ELCA	87.7 (66.9–106.3)
After ballooning	121.2 (93.3–152.9)

Mean reference vessel area (mm^3^/mm)	
After manual thrombectomy	5.4 ± 2.0
After ELCA	6.0 ± 2.0
After ballooning	7.3 ± 2.5

Minimal lumen area (mm^2^)	
After manual thrombectomy	1.4 ± 0.7
After ELCA	2.4 ± 0.9
After ballooning	5.6 ± 2.0

Culprit lesion morphology	
Ruptured plaque	58 (63.0%)
Erosion	23 (25.0%)
Calcified nodule	7 (7.6%)
Unidentified	4 (4.3%)

Predominant type of thrombus	
Red thrombus	60 (65.2%)
White thrombus	18 (19.6%)
Mixed thrombus	14 (15.2%)

Data are presented as median (interquartile range), mean ± SD, or *n* (%). ELCA = excimer laser coronary angioplasty.

**Table 4:** Comparison between ruptured and nonruptured plaque.

	Ruptured plaque (*n* = 58)	Nonruptured plaque (*n* = 31)	*p* value
Predominant type of thrombus			0.001
Red thrombus	44 (75.9%)	13 (43.3%)	
White thrombus	5 (8.6%)	12 (40.0%)	
Mixed thrombus	9 (15.5%)	5 (16.7%)	

Thrombus length (mm)	7.1 ± 3.1	6.9 ± 4.2	0.532

Thrombus volume (mm^3^)			
After manual thrombectomy	70.4 (47.8–89.8)	49.5 (33.9–70.0)	0.035
After ELCA	46.2 (30.4–62.8)	42.2 (26.0–60.9)	0.495
After ballooning	14.4 (3.9–28.0)	12.7 (4.5–31.7)	0.945

Reduction of thrombus volume by ELCA	17.5 (8.9–30.1)	8.5 (0.7–17.7)	0.004

Luminal volume (mm^3^)			
After manual thrombectomy	69.5 (52.3–90.4)	70.6 (51.4–80.4)	0.676
After ELCA	91.5 (70.7–107.7)	79.4 (59.9–92.9)	0.068
After ballooning	128.2 (102.8–160.9)	106.4 (79.8–123.9)	0.014

Minimal lumen area (mm^2^)			
After manual thrombectomy	1.4 ± 0.7	1.4 ± 0.7	0.996
After ELCA	2.5 ± 1.0	2.3 ± 0.8	0.588
After ballooning	5.8 ± 1.9	5.0 ± 1.9	0.034

Data are presented as *n* (%), median (interquartile range), or mean ± SD. ELCA = excimer laser coronary angioplasty.

## Data Availability

Data are available on reasonable request due to privacy/ethical restrictions.
